# A Novel Trypsin Inhibitor-Like Cysteine-Rich Peptide from the Frog *Lepidobatrachus laevis* Containing Proteinase-Inhibiting Activity

**DOI:** 10.1007/s13659-015-0069-z

**Published:** 2015-09-02

**Authors:** Yu-Wei Wang, Ji-Min Tan, Can-Wei Du, Ning Luan, Xiu-Wen Yan, Ren Lai, Qiu-Min Lu

**Affiliations:** Life Sciences College of Nanjing Agricultural University, Nanjing, 210095 Jiangsu China; Key Laboratory of Animal Models and Human Disease Mechanisms of Chinese Academy of Sciences & Yunnan Province, Kunming Institute of Zoology, Kunming, 650223 Yunnan China

**Keywords:** Trypsin inhibitor, Cysteine-rich peptide, Amphibian, Skin

## Abstract

**Abstract:**

Various bio-active substances in amphibian skins play important roles in survival of the amphibians. Many protease inhibitor peptides have been identified from amphibian skins, which are supposed to negatively modulate the activity of proteases to avoid premature degradation or release of skin peptides, or to inhibit extracellular proteases produced by invading bacteria. However, there is no information on the proteinase inhibitors from the frog *Lepidobatrachus laevis* which is unique in South America. In this work, a cDNA encoding a novel trypsin inhibitor-like (TIL) cysteine-rich peptide was identified from the skin cDNA library of *L. laevis*. The 240-bp coding region encodes an 80-amino acid residue precursor protein containing 10 half-cysteines. By sequence comparison and signal peptide prediction, the precursor was predicted to release a 55-amino acid mature peptide with amino acid sequence, IRCPKDKIYKFCGSPCPPSCKDLTPNCIAVCKKGCFCRDGTVDNNHGKCVKKENC. The mature peptide was named LL-TIL. LL-TIL shares significant domain similarity with the peptides from the TIL supper family. Antimicrobial and trypsin-inhibitory abilities of recombinant LL-TIL were tested. Recombinant LL-TIL showed no antimicrobial activity, while it had trypsin-inhibiting activity with a *Ki* of 16.5178 μM. These results suggested there was TIL peptide with proteinase-inhibiting activity in the skin of frog *L. laevis*. To the best of our knowledge, this is the first report of TIL peptide from frog skin.

**Graphical Abstract:**

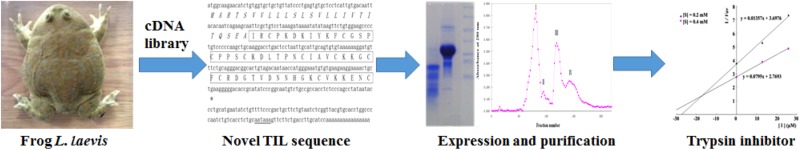

## Introduction

Amphibian skin has great molecular diversity and is a versatile organ important for everyday survival. Amphibian skin secretions are a subject of interest because of the skin’s unique chemical properties and biosynthesis pathways and because of its potential clinical applications [1]. Over the past several decades, bioactive components of amphibian skin secretions, especially biologically active peptides, have been extensively studied [2–6]. More than 100 families of peptides including about 2000 from amphibian skin have been described [1].

Many protease inhibitor peptides smaller than 10 kDa have been identified from amphibian skins [7–11]. These include bi-functional peptides with both protease-inhibitory and antimicrobial functions. Protease inhibitor peptides are categorized broadly based on structural motifs or the protease inhibited. Using the presence of a defined structural motif, these peptides can be classified into Kunitz, Kasal, and Bowman-Birk classes; by inhibited protease, they are classified into cystatins, serpins, and tissue inhibitors of metalloproteases [10, 12]. However, there are rare reports of trypsin inhibitor-like cysteine rich (TIL) peptides from amphibian skin.

TIL domain typically contains ten cysteine residues that form five disulfide bridges. The cysteine residues that form the disulfide bridges are C1–C7, C2–C6, C3–C5, C4–C10 and C8–C9 [13]. Besides the trypsin inhibitors, it is interesting that many extracellular proteins also contain multiple TIL domains. Peptides containing the TIL domain generally consist of 56–84 amino acid residues. It was shown that a typical peptide of the TIL family is able to inhibit proteinase, and thus play roles in biological processes, such as inhibition of anticoagulation and participation in immune response [14–17]. Because TIL domain-containing peptides exhibit some unique features, such as compact rigidity, well-defined structure and small size, they are used as a molecular scaffold for the development of new pharmaceutical drugs [18].

However, only a small number of the peptides containing TIL domain have been identified so far, which hinders the structure–function relationship investigation, evolutionary analysis and potential uses of this kind of peptides [18]. In this work we identified a TIL peptide from the skin of from the frog *L. laevis* and conformed its proteinase-inhibiting activity.

## Results and Discussion

### Cloning of LL-TIL Precursor

**Fig. 1:**
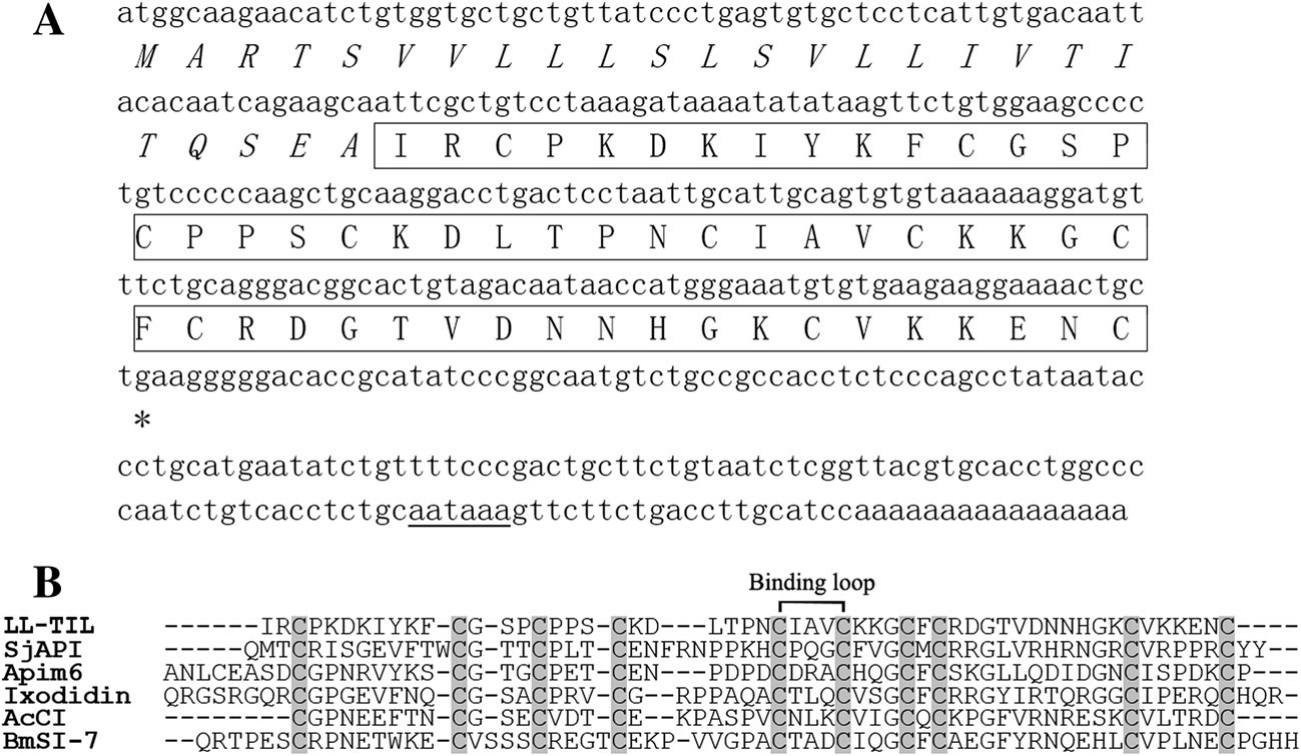
The nucleotide sequence encoding LL-TIL precursor (**a**) and the precursor sequence alignment of LL-TIL with SjAPI (Q8T0W5), Apim6 (NP_001035360), Ixodidin (P83516), AcCI (AGB06350) and BmSI-7 (ACV83329) (**b**). The signal peptide is *italic*. The sequence of mature peptide is *boxed*. The *asterisk* (*) indicates the stop codon. The polyA signal (AATAAA) is* underlined*. The conserved cysteine motifs are highlighted in *grey*. The *bar* (-) was introduced for optimal comparison

As illustrated in Fig. 1a, a cDNA encoding an 80-amino acid residue (aa) precursor protein was cloned from the skin cDNA library of the frog *L. laevis*. The precursor includes a predicted signal peptide and mature peptide (named LL-TIL). Mature LL-TIL is composed of 55 aa including 10 half-cysteines. MW/pI tool (http://www.expasy.ch/tools/pi_tool.html) showed that it had a predicted molecular mass of 6164.29 and an isoelectric point (pI) of 8.88.

BLAST search revealed that the sequence of LL-TIL showed significant domain similarity to arthropod peptides from the TIL supper family including SjAPI (Q8T0W5), Apim6 (NP_001035360), Ixodidin (P83516), AcCI (AGB06350) and BmSI-7 (ACV83329) as illustrated in Fig. 1b. SjAPI and Apim6 are produced and secreted by the venom glands of the scorpion or bee venom [14, 16, 19]. SjAPI potently inhibits the activities of α-chymotrypsin and elastase [14]. Api m6 is a bee venom allergen that binds to IgE [16]. Ixodidin is from hemocytes of the cattle tick [15]. It is able to inhibit the activities of chymotrypsin and elastase. It also possesses weak antimicrobial activities and is involved in immune response. AcCI is from the honeybee [20]. It inhibits the activities of chymotrypsin and elastase. BmSI-7 (ACV83329) is expressed in the eggs, ovary, gut, salivary gland and hemocytes of tick [17]. BmSI-7 inhibits the activity of subtilisin, and might participate in defense and inflammatory response. Although the primary structures of these peptides vary, they share a common cysteine motif: CX8–9CX3–5CX3–4CX6–10CX3–6 CX3–5 CX1CX11–13CX5C (X stands for any amino acid residue). There is a protease-binding loop located between C5 and C6 in the TIL peptides (Fig. 1b). The three residues are the active site residues that affect the activities and specificities of the peptides [13–17]. According to the residues at the proteinase-binding loop, LL-TIL was predicted to have trypsin- or chymotrypsin-inhibiting activity. In order to verify the biological activity of LL-TIL, it was expressed as a recombinant peptide.

### Expression and Purification of Recombinant LL-TIL

**Fig. 2:**
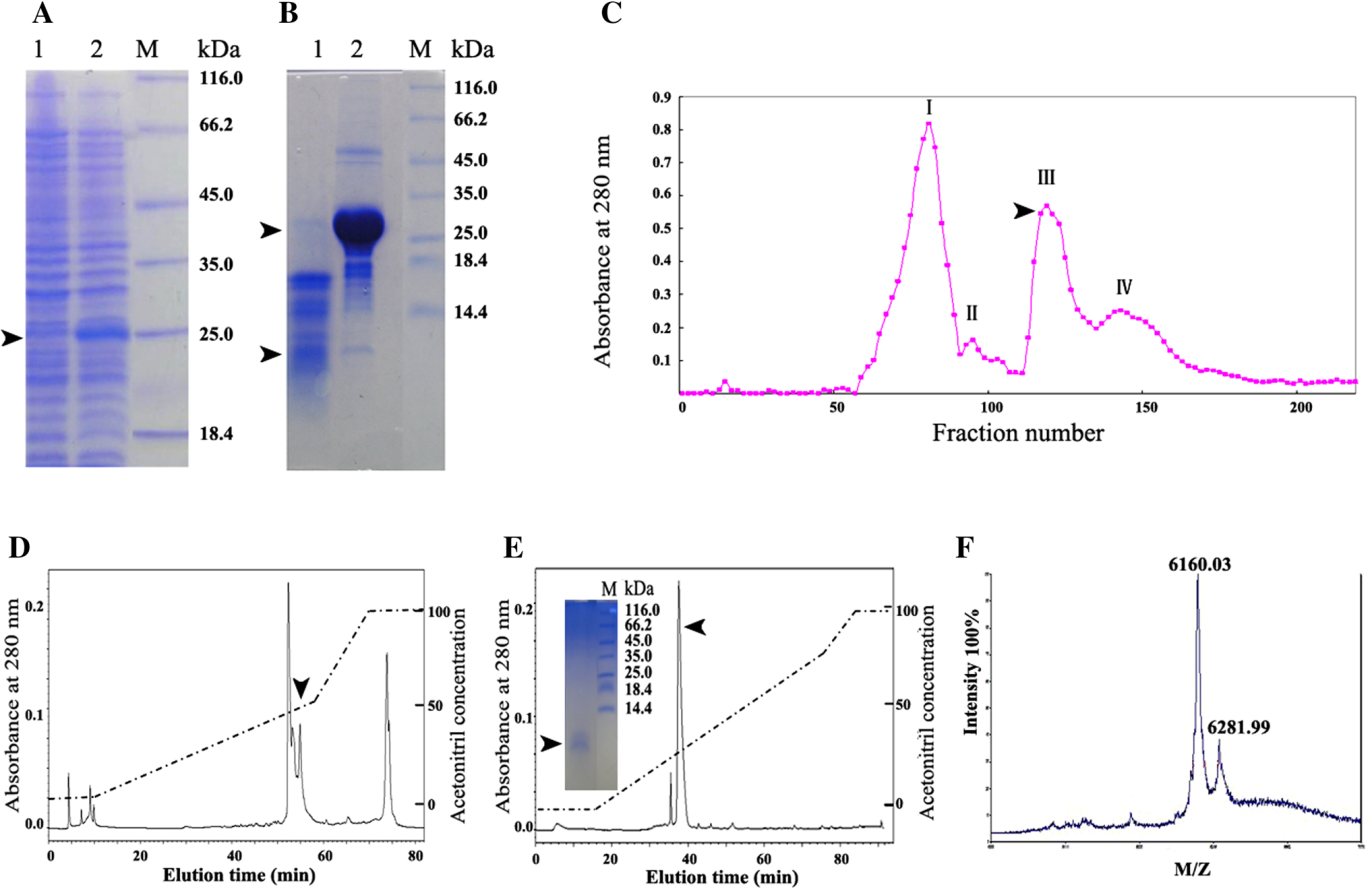
Expression and purification of LL-TIF fusion protein in *E. coli* Rosetta-gami (DE3). **a** Expression of LL-TIL fusion protein in *E. coli* Rosetta-gami (DE3) and analyzed by SDS-PAGE (12 %). M, protein molecular weight marker;* lane 1*, LL/pET-32a (+) uninduced; *lane 2*, LL/pET-32a (+) induced supernatant. The target molecular weight is indicated by an *arrow*. **b** SDS-PAGE (15 %) analysis of eluted LL-TIL fusion protein from His-binding resin Ni^2+^ affinity column (*lane 2*) and its digested products by formic acid (*lane 1*). The fusion protein and the released LL-TIF are indicated by* arrows* (up for the fusion protein and low for the released LL-TIF, respectively). **c** Sephadex G-50 chromatography of digested products of LL-TIF fusion protein equilibrated with 0.1 M Na_2_HPO_4_–NaH_2_PO_4_. The elution was performed at a flow rate of 0.3 mL/min with the same buffer. **d** The protein peak III (indicated by an *arrow*) containing trypsin-inhibiting activity was further purified on a Hypersil C_4_ RP-HPLC column equilibrated with 0.1 % (v/v) trifluoroacetic acid in water. The elution was performed with the gradients of acetonitrile in 0.1 % (v/v) trifluoroacetic acid in water at a flow rate of 0.7 mL/min. Fractions containing trypsin-inhibiting activity is indicated by an *arrow*. **e** The recombinant LL-TIF was further purified on the Spherisob C_8_ RP-HPLC column. Fractions containing trypsin-inhibiting activity is indicated by an *arrow*. **f** Mass spectrum analysis of purified recombinant LL-TIF on a MALDI-TOF mass spectrometer. *Inserts* in **e** indicate SDS-PAGE (15 %) of purified LL-TIF

The expression of recombinant LL-TIL was induced by IPTG (Fig. 2a). The fusion protein (Fig. 2a) isolated from the His-binding resin Ni^2+^ affinity column was cut by 50 % formic acid (50 °C, 24 h) (Fig. 2b). The mature LL-TIL was released from the fusion protein, and then purified by Sephadex G-50 size-exclusion chromatography (Fig. 2c), C_4_ and C_8_ RP-HPLC (Fig. 2d, e) columns. MALDI-TOF mass spectrometry analysis gave a mass of 6160.03 (Fig. 2f) that is accord with the predicted molecular weight. The homogeneity of the purified recombinant LL-TIL was confirmed by SDS-PAGE and mass spectrometry analysis (Fig. 2e, f). The first 15 amino acids of the N-terminal sequence of IL-TIL was further confirmed by Edman degradation sequencing to be the same as the sequence deduced from its cDNA.

### Antimicrobial Activities

Recombinant LL-TIL showed no antimicrobial abilities against the tested microorganisms including Gram positive bacterium *Staphylococcus aureus* (ATCC 25923), Gram-negative bacteria *Escherichia coli* (ATCC 25922), *Bacillus subtilis* (ATCC 6633) and fungus *Candida albicans* (ATCC 20032) (Data not shown).

### Proteinase-Inhibiting Activity

**Fig. 3:**
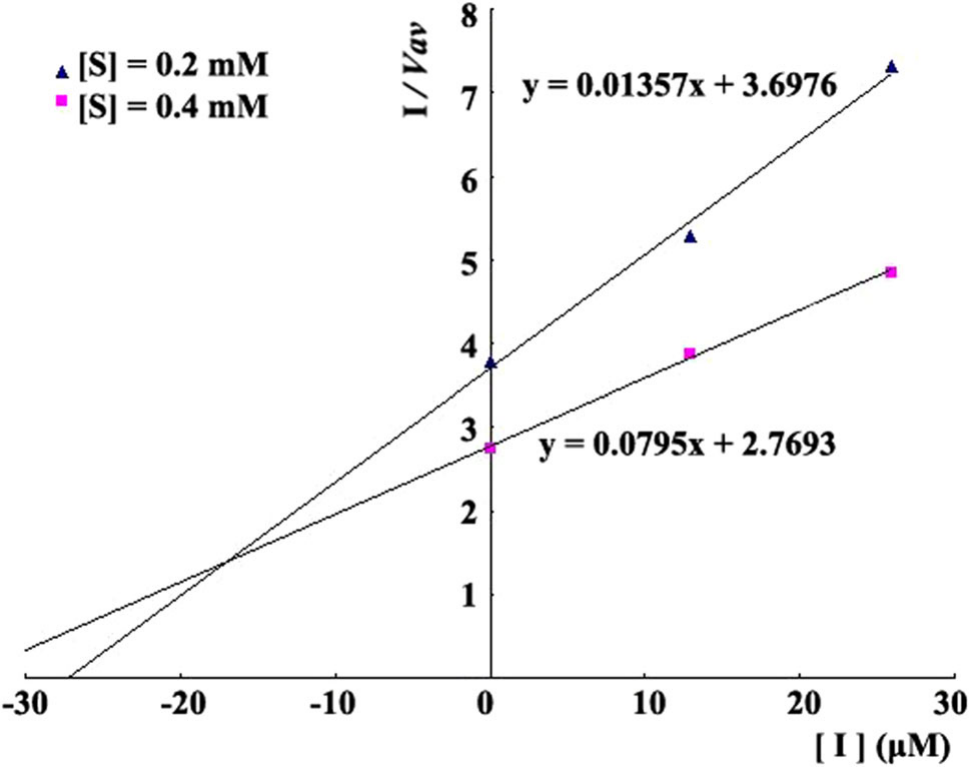
The *Ki* of LL-TIF on trypsin is 16.5178 μM by Dixon plot

LL-TIL did not inhibit the hydrolysis of synthetic chromogenic substrates by thrombin, chymotrypsin, elastase, plasmin and substilisin (Data not shown). However, it inhibited the activity of trypsin. The reaction velocity of trypsin with chromogenic substrate in the presence of LL-TIL was shown in Table 1. By Dixon plot (Fig. 3), the *Ki* was calculated to be 16.5178 μM. Compared with Bowman − Birk-like trypsin inhibitors from frog [8], the activity of LL-TIL is lower than Ranacyclin B3, -B5, and -B-RL1 which had trypsin-inhibitory abilities with a *Ki* order of 10^−8^ M. Its activity is similar to Ranacyclin-B-RN1, -B-RN2, -B-RN6, -B-LK1, and -B-LK2 which had a *Ki* order of 10^−6^ M. However, its activity is much high than Ranacyclin-B-AL1 which had weak trypsin-inhibitory activity with a *Ki* of 10^−4^ M. Besides, its activity is lower than the Kunitz inhibitors from European frog *Bombina bombina*, which had *Ki* values in the range of 0.1–1 μM [21]. Presently, the biological role of proteinase inhibitory peptides in amphibian skins is explained mainly by two hypotheses. One is that several enzymes are involved in peptide precursor processing or peptide degradation in the skin and proteinase inhibitors negatively modulate the activity of these proteases to avoid premature degradation or release of skin peptides [22]. The other hypothesis is that the peptides are surface anti-infective agent similar to AMPs because they inhibit extracellular proteases produced by invading bacteria [21]. LL-TIL showed trypsin-inhibiting activity, but its biological target and function are not known. Further work is needed to address this issue.

**Table 1:** Reaction velocities of trypsin with substrate at different concentrations in the presence of LL-TIL (I)

[I] μM	[S] = 0.2 mM		[S] = 0.4 mM	
	*V*_*av*_	1/*V*_*av*_	*V*_*av*_	1/*V*_*av*_
0	0.2641	3.7864	0.3656	2.7352
12.987	0.1893	5.2826	0.2584	3.8700
25.974	0.1368	7.3116	0.2063	4.8481

The values are means of three duplicate experiments

## Experimental Section

### cDNA Library Construction

One adult male frog of *L. laevis* was purchased from pet market in Nanjing, Jiangsu Province of China. All applicable institutional and/or national guidelines for the care and use of animals were followed. Total RNA was extracted from the skin of the frog by TRIzol reagent (Life Technologies, Ltd.). The cDNA was synthesized using a SMART™ PCR cDNA synthesis kit purchased from Clontech (Palo Alto, CA). The primers used in the first strand synthesis were cDNA 3′ SMART CDS Primer II A, 5′-AAGCAGTGGTATCAACGCAGAGTACT (30) VN-3′ (N = A, C, G or T; V = A, G or C), and SMART II A oligonucleotide, 5′-A AGCAGTGGTATCAACGCAGAGTACGCGGG-3′. The second strand was amplified using Advantage polymerase by 5′ PCR primer II A, 5′-A AGCAGTGGTATCAACGCAGAGT-3′. A directional cDNA library was constructed with a plasmid cloning kit (SuperScript™ Plasmid System, GIBCO/BRL) following the instructions of manufacturer, producing a library of about 1.0 × 10^6^ independent colonies.

### cDNA Cloning and Construction of Recombinant Vector

More than one thousand clones were randomly selected and isolated. They are subjected to DNA sequencing by an applied biosystems DNA sequencer (ABI 3730). A clone was found to encode a precursor containing mature peptide (named LL-TIL) which shares a significant sequence similarity with the peptides from TIL supper family. *E. coli* Rosetta-gami (DE3) and plasmid pET-32a (+) were used to express the recombinant LL-TIL. A formic acid cleavage site, -(GACCCG)-, was designed at the right upstream to the LL-TIL coding sequence. Rare codons for Asp and Pro were replaced by more frequently used ones based on *E. coli* codon usage. Two forward primers: LL-5′-1 (5′-GATGATGACCCGATTCGCTGTC-3′), LL-5′-2 (5′-CGGGGTACCGATGATGATGAC-3′) and a reverse primer LL-3′ (5′-CCCAAGCTTGAGGTGGCGG-3′) were designed. LL-5′-1 contains the formic acid cleavage site. LL-5′-2 and LL-3′ contains recognition site for Kpn I and Hind III, respectively. The PCR conditions were 4 min at temperature of 95 °C, and then running 26 cycles with a temperature profile of 30 s at 94 °C, 40 s at 60.5 °C, and 50 s at 72 °C, at last holding at 72 °C for 10 min. The final purified PCR product was digested with Kpn I and Hind III, and ligated into the pET-32a (+) plasmid with T4 DNA ligase at the corresponding restriction sites. The resultant recombinant vector was referred to as LL/pET-32a (+).

### Expression of Recombinant LL-TIL

The LL/pET-32a (+) construct was transformed into the *E. coli* strain Rosetta-gami (DE3) for protein expression. After overnight growth in 1 mL LB media (with 100 μg/mL ampicillin and 12.5 μg/mL tetracycline) at 37 °C, this bacterial culture was used to inoculate another 1000 mL LB media. When the absorbance at 600 nm was reached 0.6, isopropyl β-d-1-Thiogalactopyranoside (IPTG) (final concentration of 0.8 mM) was added to initiate fusion protein expression by culturing at 28 °C. Following additional 3 h cultivation, cells were harvested by centrifugation at 10000×*g* for 15 min. The histidine-tagged carrier and residual undigested fusion protein were released from the cell by sonication, and applied on a His-binding resin Ni^2+^ affinity column (Merck). The LL-TIL-containing fusion protein was hydrolyzed by formic acid (50 %) at 50 °C for 24 h according to the manufacturer’s instructions. The reaction mixture was then lyophilized.

### Purification of Recombinant LL-TIL

Lyophilized sample containing recombinant LL-TIL (200 mg) was dissolved in 2 mL of 0.1 M Na_2_HPO_4_–NaH_2_PO_4_, pH 6.0. And then applied to a Sephadex G-50 size-exclusion chromatography column (1000 × 26 mm) equilibrated with 0.1 M Na_2_HPO_4_–NaH_2_PO_4_, pH 6.0. The elution was performed at a flow rate of 0.3 mL/min with the same buffer. The absorbance of the eluate was monitored at 280 nm. Every eluted fraction (3 mL) was collected and subjected to antimicrobial and trypsin inhibitory assays as described below. Fractions containing trypsin-inhibiting activity were pooled, lyophilized, and re-suspended in 5 mL 0.1 M Na_2_HPO_4_–NaH_2_PO_4_, pH 6.0 for purification by a C_8_ reverse phase high-performance liquid chromatography (RP-HPLC, Hypersil C_4_, 25 × 0.46 cm) column. The LL-TIL containing fraction was lyophilized and further purified with the Spherisob C_8_ column on HPLC. The purity of the peptide was determined by SDS–polyacrylamide gel electrophoresis (SDS-PAGE) and mass spectrometry analysis.

### SDS-PAGE and Protein Concentration Determination

SDS-PAGE was performed under reduced and/or non-reduced conditions. Protein samples were loaded onto 12 or 15 % polyacrylamide gels. Protein bands were visualized after using a standard Comassie blue stain. The protein concentration was determined by a protein assay kit (Bio-Rad, Hercules, CA) using bovine serum albumin (BSA) as a standard.

### Mass Spectrometry

Mass spectrometry analysis was performed on a matrix-assisted laser desorption ionization time-of-flight mass spectrometer (MALDI-TOF–MS) AXIMA CFR (Kratos Analytical). α-Cyano-4-hydroxycinnamic acid (CHCA) was used as matrix. The specific parameters were as follows: the ion acceleration voltage was 20 kV, the accumulating time of single scanning was 50 s, polypeptide mass standard (Kratos Analytical) serving as external standard.

### Serine Protease Inhibitory Testing

Effects of the tested samples on the hydrolysis of synthetic chromogenic substrates by different serine proteases were assayed in 0.05 M Tris–HCl, pH 7.8 (for trypsin, Sigma T4665; thrombin, Sigma T4393; chymotrypsin, Sigma C4129; elastase, Sigma E1250; plasmin, Sigma P1867) or pH 8.45 (for substilisin, Sigma P5380) buffer at 37 °C. The protease (trypsin, 10 nM; thrombin, 10 nM; chymotrypsin, 21 nM; elastase, 23 nM and substilisin, 30 nM) and different amounts of LL-TIL (final concentrations ranging from 0.0 to 20 μM) were pre-incubated for 10 min at 37 °C. The reaction was initiated by the addition of the substrate B-3133 (*N*-benzyl-l-arginine-4-nitroanilide-hydrochride-*p*NA, Sigma) (0.5 mM). The formation of *p*-nitroaniline was monitored continuously at 405 for 2 min. For chymotrypsin, the reaction was monitored at 253 nm. The inhibition constant *Ki* was determined according to the reported method [23].

### Antimicrobial Assays

For antimicrobial assays, several microorganisms including Gram positive bacterium *S. aureus* (ATCC 25923), Gram-negative bacteria *E. coli* (ATCC 25922), *B. subtilis* (ATCC 6633), and fungus *C. albicans* (ATCC 20032) were obtained from Kunming Medical College. They were first grown in LB (Luria–Bertani) broth or yeast extract–peptone–dextrose broth as our previous methods [24]. MIC (minimal inhibitory concentration) of tested sample against these microorganisms was determined as previous reports [24]. The MIC is defined as the lowest concentration of tested sample to inhibit microorganism growth.

## Conclusion

This work identified and characterized a novel trypsin inhibitor-like cysteine-rich (TIL) peptide in the frog of *L. laevis*. Its biological activities were investigated. LL-TIL showed potential proteinase inhibiting activity with no antimicrobial activity. Though its biological target and function are elusive, it may negatively modulate the activity of proteases to avoid premature degradation or release of skin peptides, or inhibit extracellular proteases produced by invading bacteria. This is the first report of TIL peptide with proteinase inhibiting activity in frog. LL-TIL may be useful in the studies of the structure–function relationship, evolutionary analysis and potential uses of TIL peptides.
